# Two-component carnitine monooxygenase from *Escherichia coli*: functional characterization, inhibition and mutagenesis of the molecular interface

**DOI:** 10.1042/BSR20221102

**Published:** 2022-09-21

**Authors:** Fabian Piskol, Kerstin Neubauer, Maurice Eggers, Lisa Margarete Bode, Jan Jasper, Alan Slusarenko, Edward Reijerse, Wolfgang Lubitz, Dieter Jahn, Jürgen Moser

**Affiliations:** 1 Institute of Microbiology, Technical University Braunschweig, Braunschweig, Germany; 2 Institut für Biologie III, RWTH Aachen University, Aachen, Germany; 3 Max-Planck-Institute for Chemical Energy Conversion, Mülheim an der Ruhr, Germany; 4 Braunschweig Centre of Integrated Systems Biology, Braunschweig, Germany

**Keywords:** l-carnitine, Carnitine monooxygenase, gut microbiota, Rieske-type oxygenase, site-directed mutagenesis, trimethylamine

## Abstract

Gut microbial production of trimethylamine (TMA) from l-carnitine is directly linked to cardiovascular disease. TMA formation is facilitated by carnitine monooxygenase, which was proposed as a target for the development of new cardioprotective compounds. Therefore, the molecular understanding of the two-component Rieske-type enzyme from *Escherichia coli* was intended. The redox cofactors of the reductase YeaX (FMN, plant-type [2Fe-2S] cluster) and of the oxygenase YeaW (Rieske-type [2Fe-2S] and mononuclear [Fe] center) were identified. Compounds meldonium and the garlic-derived molecule allicin were recently shown to suppress microbiota-dependent TMA formation. Based on two independent carnitine monooxygenase activity assays, enzyme inhibition by meldonium or allicin was demonstrated. Subsequently, the molecular interplay of the reductase YeaX and the oxygenase YeaW was addressed. Chimeric carnitine monooxygenase activity was efficiently reconstituted by combining YeaX (or YeaW) with the orthologous oxygenase CntA (or reductase CntB) from *Acinetobacter baumannii*. Partial conservation of the reductase/oxygenase docking interface was concluded. A structure guided mutagenesis approach was used to further investigate the interaction and electron transfer between YeaX and YeaW. Based on AlphaFold structure predictions, a total of 28 site-directed variants of YeaX and YeaW were kinetically analyzed. Functional relevance of YeaX residues Arg^271^, Lys^313^ and Asp^320^ was concluded. Concerning YeaW, a docking surface centered around residues Arg^83^, Lys^104^ and Lys^117^ was hypothesized. The presented results might contribute to the development of TMA-lowering strategies that could reduce the risk for cardiovascular disease.

## Introduction

The human gut contains a myriad of microbes that are involved in the metabolism of food constituents [1]. The metabolic products of these microbial consortia also play an important role in the development of certain diseases [2–5]. This was exemplified by the bacterial metabolite trimethylamine (TMA), which is produced by various taxa of the gut microbiota from dietary nutrients that contain a TMA moiety, e.g., l-carnitine or choline.

Nutritional l-carnitine originates essentially from meat or milk products, but it is also found in dietary supplements aimed at reducing adipose tissue during exercise. Consumption of large amounts of meat results in a higher plasma l-carnitine concentration, which is not observed in vegetarians. Main dietary sources of choline are egg yolk, liver and various meats. Adults that regularly consume meat acquire 80–190 mg l-carnitine [6] and ∼400 mg choline per day [7].

TMA is absorbed via the intestinal epithelium and is then further oxidized in the human liver to trimethylamine N-oxide (TMAO) by flavin monooxygenases (FMOs). Increased TMAO levels were associated with severe cardiovascular disease and atherosclerosis [8–10]. It was proposed that TMAO promotes atherosclerotic plaque formation by exacerbating vascular inflammation, impairing vascular functions and disturbing cholesterol homeostasis [11]. Furthermore, TMAO has recently emerged as a candidate risk factor for metabolic dysfunction-associated fatty liver disease [12].

**Figure 1:**
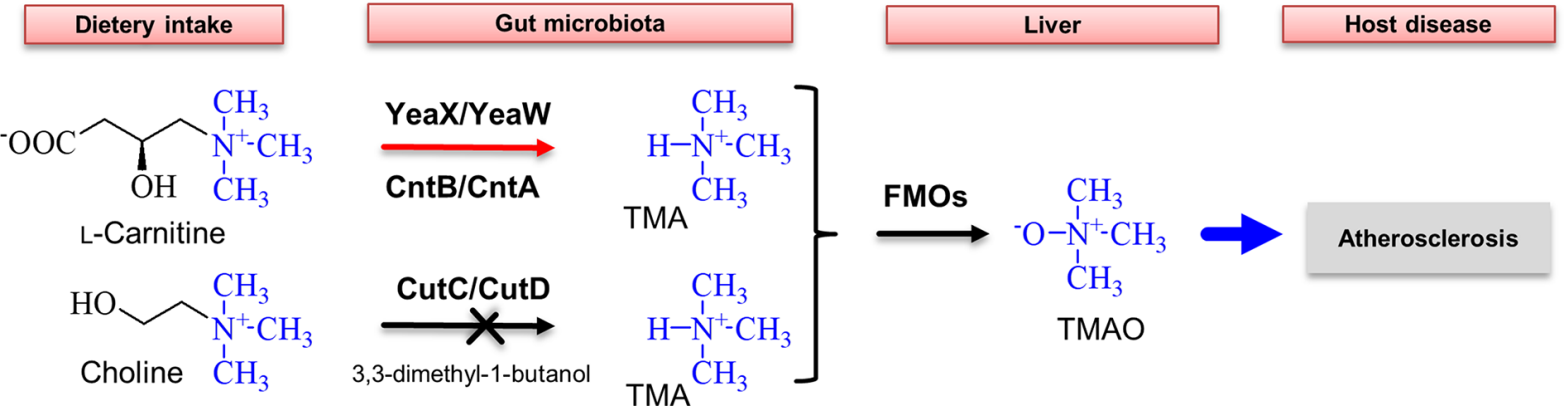
Metabolism of l-carnitine and choline is associated with atherosclerosis development The gut microbiome facilitates the metabolism of dietary nutrients l-carnitine or choline. Cleavage of the C–N bond is catalyzed by TMA lyases YeaX/YeaW (termed CntB/CntA in *A. baumannii*) or CutC/CutD. TMA is further converted by FMOs of the liver. The resulting TMAO molecule enhances the development of atherosclerosis and is associated with the risk for myocardial infarction and stroke. Targeted inhibition of CutC/CutD efficiently prevented the formation of TMA in the mouse model.

Possible intervention strategies to reduce circulating TMA concentrations might include a diet which avoids excessive amounts of TMA precursors (e.g. l-carnitine or choline from red meat or eggs). Fish is rich in TMAO and thus has an unfavorable influence on TMAO levels. Furthermore, it was demonstrated that the individual composition of the intestinal flora is an important determinant for the production of TMA [12]. Accordingly, manipulation of the gut microbiota was proposed as a therapeutic strategy to circumvent cardiovascular disease [13]. The C–N bond of dietary choline is cleaved by the oxygen-independent enzyme choline trimethylamine-lyase (CutC/CutD) into TMA and the central metabolite acetaldehyde [14,15]. Targeted inhibition of CutC/CutD by a substrate analog 3,3-dimethyl-1-butanol was shown to attenuate choline diet-enhanced atherosclerosis in a mouse model [16] (Figure 1).

**Figure 2:**
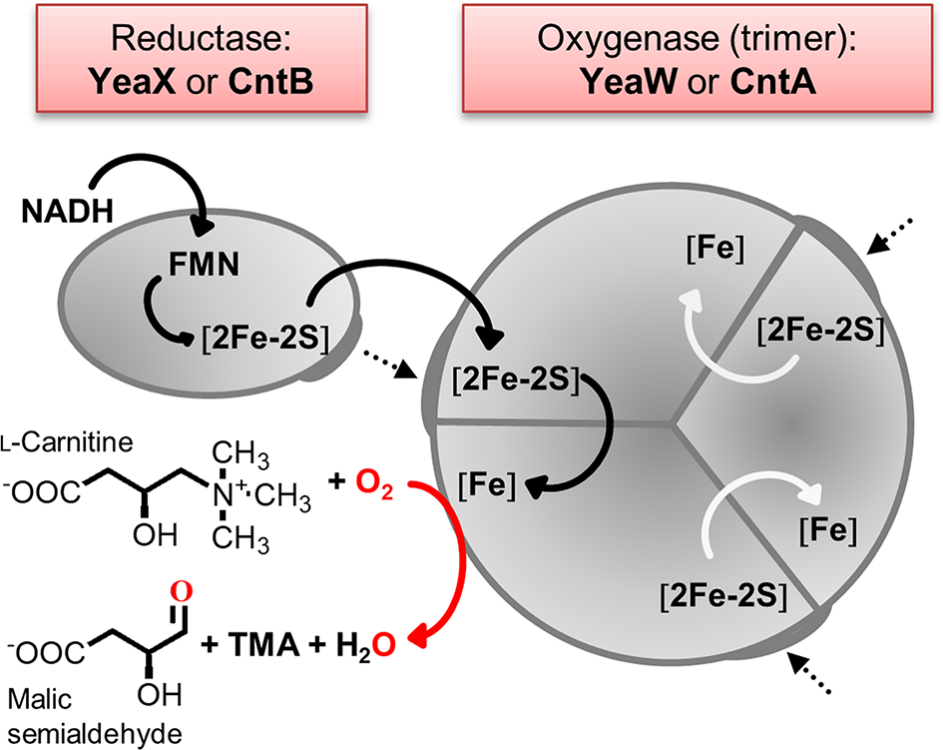
Redox catalysis of carnitine monooxygenase The reductase YeaX from *E. coli* (termed CntB in *A. baumannii*) delivers electrons from NADH via a plant-type [2Fe-2S] cluster and FMN onto the oxygenase component YeaW (termed CntA in *A. baumannii*). Trimeric YeaW contains a Rieske-type [2Fe-2S] cluster and a mononuclear [Fe] center. YeaW allows for the electron transfer between the Rieske-type [2Fe-2S] cluster and the [Fe] of two adjacent monomers. Reductive activation of O_2_ initiates the monooxygenation of l-carnitine, which subsequently results in the formation of malic semialdehyde and TMA.

Carnitine monooxygenase CntB/CntA (EC 1.14.13.239) facilitates the oxygen-dependent cleavage of l-carnitine into TMA and malic semialdehyde (Figure 2). The aldehyde serves as a carbon and energy source which is entering the tricarboxylic acid cycle by virtue of malic semialdehyde dehydrogenase and malate dehydrogenase [17]. The two-component carnitine monooxygenase enzyme is composed of the reductase CntB, which delivers electrons to the catalytic unit CntA which belongs to the large family of Rieske-type oxygenases [18]. Iron-dependent Rieske oxygenases typically comprise a specific reductase, a catalytic oxygenase unit, and possibly an additional ferredoxin component [19]. CntA is a member of the quaternary amine oxidizing group V of Rieske oxygenases [20].

Only recently our laboratory contributed the biochemical characterization of the CntB/CntA system from *Acinetobacter baumannii* [21]. For the reductase CntB, the protein ligands of a redox active flavin mononucleotide (FMN) and of a plant-type [2Fe-2S] cluster were elucidated. Concerning CntA, the ligands of a Rieske-type [2Fe-2S] cluster and of a mononuclear [Fe] center were identified. Individual redox states for the electron transfer between CntB and CntA were determined by electron paramagnetic resonance (EPR) spectroscopy. Electrons from NADH are transferred to the plant-type [2Fe-2S] cluster via the FMN of CntB. Single turnover experiments revealed the translocation of electrons on to the Rieske-type [2Fe-2S] cluster and the [Fe] center of CntA [21]. The key steps of CntB/CntA catalysis were recently supported by the 3D structure of CntA [20]. *A. baumannii* CntA exists in a head-to-tail trimeric structure that allows for the electron transfer between the Rieske-type [2Fe-2S] cluster and the [Fe] of two adjacent monomers (Figure 2). Reductive activation of O_2_ can take place at the [Fe] center and allows for the monooxygenation of l-carnitine, which subsequently results in the heterolytic cleavage of the C–N bond of the reaction intermediate [17,20,21]. The resulting malic semialdehyde serves as a carbon and energy source as it is converted into malate which then enters the central tricarboxylic acid cycle [17].

Orthologous CntA proteins (from *Escherichia coli*, *Providenzia rettgeri*, *Serratia marcescens*, *Klebsiella pneumoniae*) revealed no significant differences in their substrate specificity as indicated by high activity in the presence of l-carnitine and γ-butyrobetaine, medium activity toward glycine betaine, and very low activity in the presence of choline [22]. CntA also shows a high degree of sequence conservation when compared with distantly related Rieske-type proteins with an unrelated substrate profile (e.g. 25% identity to naphthalene 1,2-dioxygenase from *Pseudomonas putida*). Thus, sophisticated bioinformatics methods were required for the correct annotation of genes from gut microbial genomes [17,23]. Among gut reference species, CntA genes were identified in *Actinobacteria* (e.g. *Corynebacterium ammoniagenes*), *Firmicutes* (e.g. *Bacillus smithii*) and also in *Proteobacteria* (e.g. *Citrobacter freundii*) [24].

Carnitine monooxygenase is a promising drug target as the metabolism of l-carnitine in the gut microbiome results in an increased plasma TMAO level. *Actinobacteria baumanni* CntA was used in drug library screenings which revealed a first competitive inhibitor (MMV3) with a *K*_i_ of 1.1 µM [20]. Inhibition of CntA-dependent TMA formation was followed spectroscopically by quantifying the CntB-dependent NADH oxidation. In this context, the understanding of the (dynamic) interplay of subunits CntB and CntA might be relevant for the further development of inhibition strategies. Hampering the CntB/CntA protein–protein interaction or the inactivation of the reductase CntB might disturb the intermolecular electron transfer processes of carnitine monooxygenase.

*A. baumanni* is an opportunistic pathogen and therefore not suitable as a primary model organism for the long term investigation of therapeutic approaches. *Escherichia coli*, on the other hand, has been used as a model system for decades and many strains of this species (e.g. also probiotic strains) are well associated with the gut microbiome. Thus, it was our aim to establish an *E. coli*-based model system for the future investigation of carnitine monooxygenase.

Here, we describe the detailed spectroscopic characterization of the carnitine monooxygenase system YeaX/YeaW from the gut microbial strain *E. coli* KO11FL. Enzyme inhibition in the presence of two putative cardioprotective substances is elucidated on the basis of complementary kinetic experiments. Furthermore, electron transfer and the interplay of subunits YeaX and YeaW are explored. Therefore, chimeric enzymes are reconstituted and an extended structure guided mutagenesis approach is performed.

## Results

### Recombinant production and purification of carnitine monooxygenase from *E. coli*

**Figure 3:**
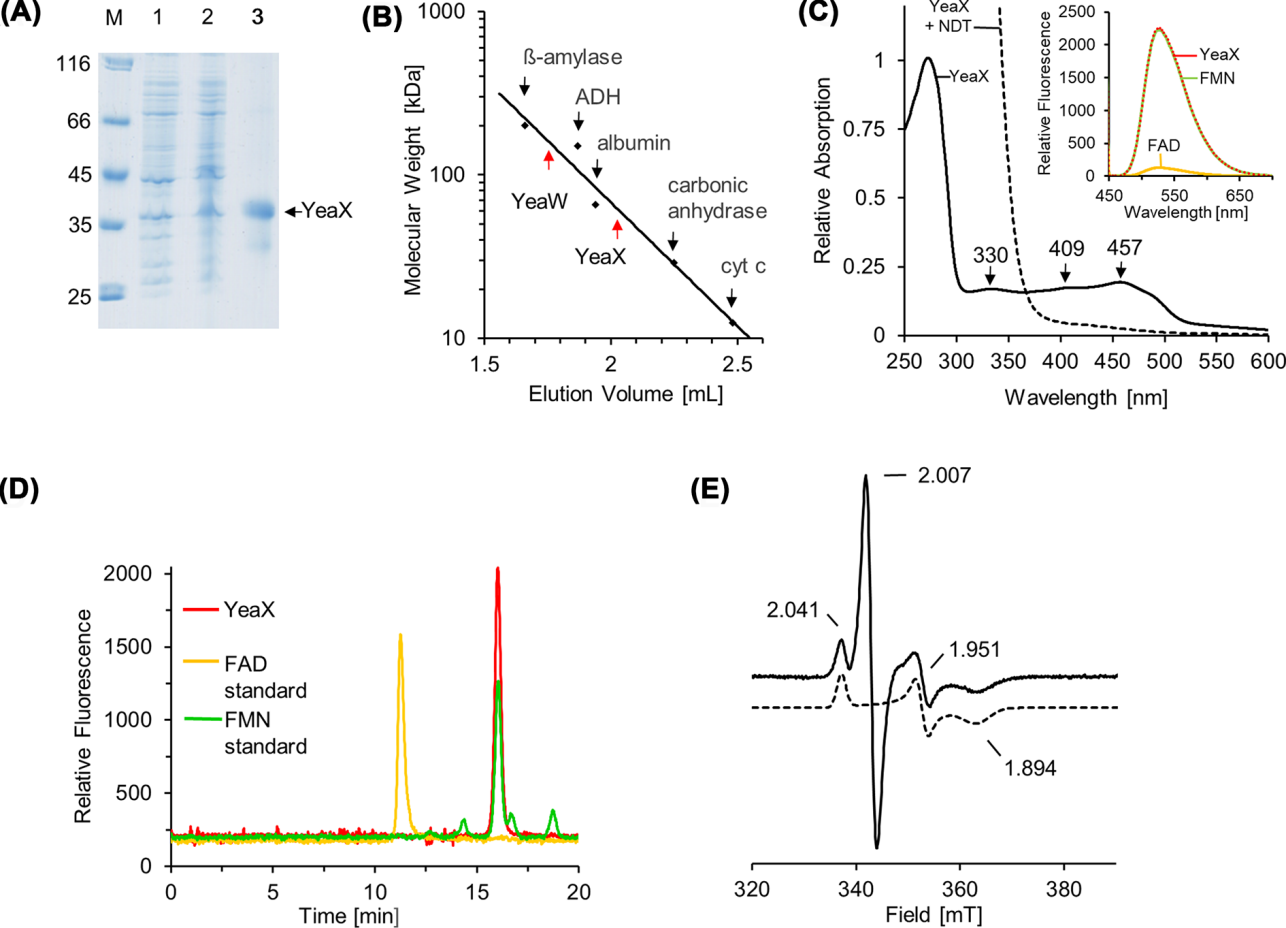
Recombinant production and characterization of YeaX (**A**) SDS-PAGE analysis of the Strep-tag purification of YeaX. Lanes 1 and 2, *E. coli* cells before and after IPTG induction; lane 3, desthiobiotin elution fraction after cell disruption, ultracentrifugation and affinity chromatography; lane M, molecular mass marker, relative molecular masses (×1000) are indicated. (**B**) Native molecular mass determination of YeaX and YeaW using analytical gel filtration. Purified proteins were analyzed on a Superdex 200 increase 5/150 GL column at a flow rate of 0.45 ml min^−1^ monitoring the absorption at 280 nm. The column was calibrated using ß-amylase (*M*_r_ = 200,000), alcohol dehydrogenase (ADH, *M*_r_ = 150,000), albumin (*M*_r_ = 66,000), carbonic anhydrase (*M*_r_ = 29,000) and cytochrome *c* (*M*_r_ = 12,400). (**C**) UV-visible spectra of purified YeaX before (black line) and after (black dashed trace) treatment with 2 mM dithionite (NDT) for 10 min (the protein spectrum is superimposed by the intrinsic absorption of dithionite in the 250–400 nm region). Inset, fluorescence spectrum of a YeaX supernatant after heat denaturation and centrifugation (370 nm excitation). The fluorescence yield of equimolar samples of FMN and FAD was analyzed as a reference. (**D**) Identification of the FMN cofactor of YeaX by HPLC. The sample from (**B**) was chromatographed on a Reprosil 100 C18 column using excitation/emission wavelengths of 370/526 nm (red). Authentic samples of FAD (yellow) or FMN (green) were analyzed accordingly. (**E**) Low temperature (15 K) EPR spectrum of a ∼1 mM YeaX sample after sodium dithionite reduction. The spectrum was recorded at 9.653 GHz, 7.5 G modulation amplitude, 0.2 mW microwave power and 100 kHz modulation frequency. The simulated spectrum is indicated as dashed line.

**Figure 4:**
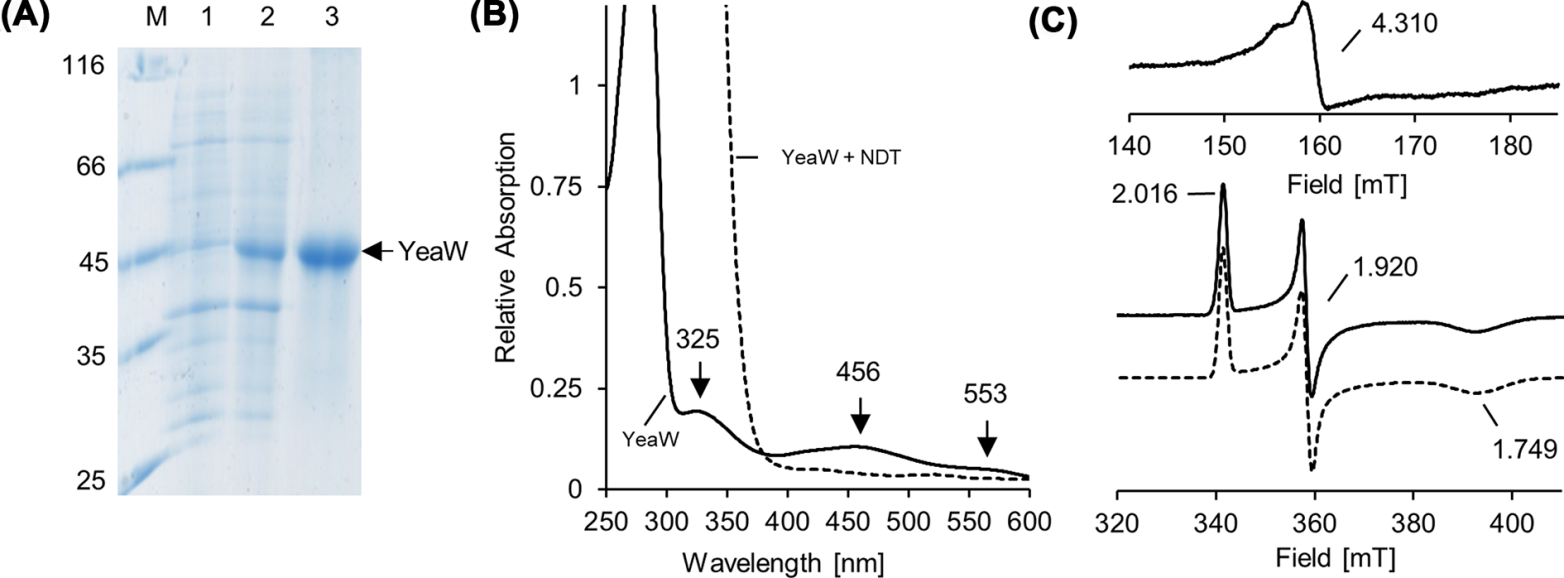
Recombinant production and characterization of YeaW (**A**) SDS-PAGE analysis of the Strep-tag purification of YeaW. Lanes 1 and 2, *E. coli* cells before and after IPTG induction; lane 3, desthiobiotin elution fractions after cell disruption, ultracentrifugation and affinity chromatography; lane M, molecular mass marker, relative molecular masses (×1000) are indicated. (**B**) UV-visible spectra of purified YeaW before (black line) and after (black-dashed trace) treatment with 10 mM dithionite for 10 min (the protein spectrum is superimposed by the intrinsic absorption of dithionite in the 250–400 nm region). (**C**) Low temperature (15 K) EPR spectra of ∼1 mM YeaW samples. Spectra were recorded at 9.653 GHz, 7.5 G modulation amplitude, 0.2 mW microwave power and 100 kHz modulation frequency. YeaW without (top) and after (bottom) dithionite reduction (10 mM). The simulated spectrum is shown as dashed line.

The genome of the gut microbial strain *E. coli* KO11FL [25] encodes for the reductase YeaX and the Rieske-type oxygenase YeaW, sharing 52 and 72% sequence identity with the related carnitine monooxygenase components CntB and CntA from *A. baumannii*. *In vitro* conversion of l-carnitine by the isolated YeaW protein has been recently demonstrated using dithionite as an artificial reductant [22]. Here, the Strep-tagged reductase YeaX and the catalytic unit YeaW were individually overproduced and purified from *E. coli* cells yielding approximately 7 and 30 mg protein from 1 l cell culture (SDS-PAGE Figures 3A and 4A, lane 3). Related purification of CntB and CntA solely revealed 5 and 6 mg per 1 l cell culture [21]. As also described for other Rieske-type proteins, components YeaX and YeaW were purified under aerobic conditions.

### Biochemical characterization of YeaX

Analytical size exclusion chromatography revealed a relative molecular mass of ∼56,000 for YeaX (38,563 Da calculated molecular mass) (Figure 3B). This might indicate that YeaX shows the running behavior of an elongated monomer as the fundamental parameter of the employed method is related to the Stokes radius and not to the molecular mass of the protein [26]. YeaX Protein samples indicated characteristic absorption maxima at 330, 409 and 457 nm (Figure 3C). Fluorescence spectroscopy of YeaX samples after heat denaturation suggested the presence of a non-covalently bound FMN cofactor (as indicated by the pH-dependent fluorescence of equimolar FAD or FMN standards) (Figure 3C, inlet) [27]. This was confirmed by HPLC analyses in the presence of authentic FAD/FMN samples (Figure 3D) and an FMN content of 1.01 ± 0.11 mol per mol YeaX was determined.

Samples of YeaX revealed 1.87 ± 0.18 mol Fe per mol protein. Characteristic bleaching of the sample was observed upon dithionite reduction (Figure 3C, dashed trace). The presence of a plant-type [2Fe-2S] cluster was verified by EPR spectroscopy. Purified YeaX (∼350 µM) was EPR silent but revealed a typical *S* = 1/2 signal with *g*-values of *g*_1_ = 2.041, *g*_2_ = 1.951, *g*_3_ = 1.894 upon sodium dithionite reduction (Figure 3E). Besides this, a dominant signal with *g* = 2.007 in the characteristic region for the semiquinone form of FMN was observed. The spin lattice relaxation of both signals was further analyzed. Increasing the sample temperature (from 15 to 85 K) revealed an almost complete loss of the plant-type [2Fe-2S]^+^ signal but did not affect the semiquinone signal. These findings clearly indicate that *E. coli* YeaX comprises FMN and a plant-type [2Fe-2S] center as redox cofactors.

### Biochemical characterization of YeaW

In analytical size exclusion chromatography experiments, the catalytic component YeaW eluted as a trimer with a relative molecular mass of ∼146,000 (45,463 Da calculated molecular mass, Figure 3B). Minor amounts of YeaW were determined as a monomer (relative molecular mass: 57,000). Functional trimer formation is of central importance for the intersubunit electron transfer of Rieske-type oxygenases [20]. Presence of an FMN or FAD cofactor was ruled out by fluorescence spectroscopy and also by HPLC analyses of heat denatured samples of YeaW (Supplementary Figure S1). Purified protein fractions of YeaW revealed absorption maxima at 325, 456 and 553 nm (Figure 4B) and an iron content of 1.92 ± 0.16 mol Fe per mol YeaW. The concentrated YeaW sample (∼500 µM) in the absence of reducing agent was subjected to EPR measurements (Figure 4C, top). A *S* = 5/2 signal of a mononuclear iron (III) center with a *g*-value of 4.310 was observed. Dithionite reduction resulted in a bleaching of the protein sample (Figure 4B, dashed trace) and the [Fe] center of YeaW was converted into the EPR silent iron (II) state [28]. In parallel, appearance of a *S* = 1/2 signal with *g*-values of *g*_1_ = 2.016, *g*_2_ = 1.920 and *g*_3_ = 1.749 (Figure 4C, bottom) was observed, which is characteristic for the presence of a [2Fe-2S]^+^ center of the Rieske-type [29]. As expected, the mononuclear iron (III) center and the [2Fe-2S]^2+^ cluster of YeaW get individually reduced by one electron in the presence of dithionite.

### Optimized l-carnitine depletion assay

**Figure 5:**
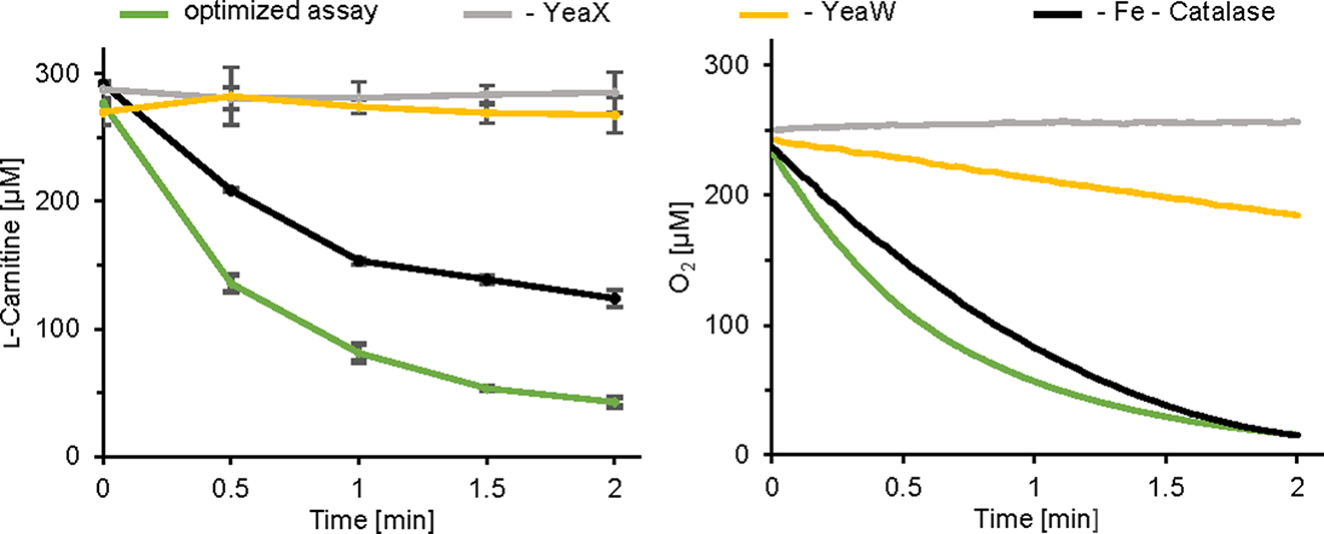
Reconstitution of carnitine monooxygenase activity Assays containing purified YeaW, purified YeaX and 300 µM l-carnitine at 27°C were initiated by addition of NADH. Optimized assay (green traces), assay in the absence of Fe^2+^ and catalase (black traces), control reaction in the absence of YeaW (yellow traces) or YeaX (gray traces). Left, l-carnitine depletion assay: Samples (prepared in triplicates) were collected after 0, 0.5, 1, 1.5 and 2 min and l-carnitine concentrations were colorimetrically quantified in a coupled carnitine acetyltransferase assay (detailed under experimental procedures). A specific activity of 1288 ± 48 nmol min^−1^ mg^−1^ was determined. Right, continuous O_2_ depletion assay (prepared in triplicate) using an optical oxygen sensor (Firesting device) resulted in a specific activity of 1506 ± 17 nmol min^−1^ mg^−1^. Only one representative graph is shown.

Enzymatic conversion of l-carnitine has been followed by ion chromatography, gas chromatography-mass spectrometry (GC-MS) based approaches [17] or by spectroscopic determination of the NADH consumption [20]. Recently, we established a microplate reader-based l-carnitine depletion assay [21]. l-Carnitine is quantified using acetyl-coenzyme A and carnitine acetyltransferase and the emerging coenzyme A is colorimetrically quantified using Ellman’s reagent (cleavage of 5,5′-dithiobis-(2-nitrobenzoic acid) into 2-thio-5-nitrobenzoic acid). To create optimal reaction conditions for carnitine monooxygenase from *E. coli* and *A. baumannii* at the same time, assay conditions were adopted appropriately. Therefore, a reaction temperature of 27°C was refined, which resulted in high specific activities of ∼47 and ∼780 nmol min^−1^ mg^−1^ for the *A. baumannii* and *E. coli* system. Substantially increased values of 353 ± 68 and 1288 ± 48 nmol min^−1^ mg^−1^ were observed after the addition of 9 µM FeSO_4_ and 360 nM catalase (Figure 5, left, compare Table 1). The same additives also resulted in an increased *in vitro* activity for a distantly related Rieske-type oxygenase (naphthalene dioxygenase from *Pseudomonas sp*. strain NCIB 9816-4) [30].

**Table 1:** Enzymatic activity of carnitine monooxygenase

Assay conditions	Components	Relative l-carnitine depletion activity (%)	Relative O_2_ depletion activity (%)
Optimized assay conditions	YeaX + YeaW	100	100
- Fe^2+^, - catalase	YeaX + YeaW	61 ± 7	77 ± 3
	YeaX	0	10 ± 2
	YeaW	0	0
	CntB + CntA	27 ± 5	24 ± 4
Meldonium			
100 µM	YeaX + YeaW	84 ± 7	107 ± 2
250 µM	YeaX + YeaW	64 ± 2	109 ± 2
500 µM	YeaX + YeaW	54 ± 9	111 ± 2
750 µM	YeaX + YeaW	40 ± 2	115 ± 1
1 mM	YeaX + YeaW	29 ± 5	120 ± 1
Fresh garlic extract			
∼ 200 µM allicin	YeaX + YeaW	33	-
∼ 2 mM allicin	YeaX + YeaW	12	-
Allicin			
10 µM (8 min)	YeaX + YeaW	93 ± 1	-
25 µM (8 min)	YeaX + YeaW	85 ± 1	-
50 µM (8 min)	YeaX + YeaW	56 ± 6	-
100 µM (8 min)	YeaX + YeaW	17 ± 3	-
Allicin			
61 µM (5 min)	YeaX + YeaW	62 ± 6	66^*^
61 µM (10 min)	YeaX + YeaW	46 ±3	45^*^
61 µM (35 min)	YeaX + YeaW	24 ± 4	30^*^
61 µM (65 min)	YeaX + YeaW	14 ± 1	23^*^
Chimeric reconstitution			
	YeaX + CntA	100 ± 4	94 ± 4
	CntB + YeaW	20 ± 2	4 ± 1

Table summarizing the optimization of assay conditions, the inhibition of the YeaX/YeaW system and the results of chimeric experiments. Carnitine monooxygenase enzymes composed of individual subunits from *E. coli* (YeaX or YeaW) and *A. baumannii* (CntB or CntA) were reconstituted under conditions of the optimized assay. The specific activity of the optimized l-carnitine depletion assay (1288 ± 48 nmol min^−1^ mg^−1^) or of the O_2_ depletion assay (1506 ± 17 nmol min^−1^ mg^−1^) were set as 100% (compare experimental procedures). -, experiment not performed; ^*^ the sensor material is effectuated in the presence of allicin, activity measurements performed as single determination.

### O_2_ depletion assay

Molecular oxygen acts as a key substrate in the reaction of carnitine monooxygenase. Accordingly, we intended to make use of a fiber optic oxygen sensor to assess the conversion of l-carnitine in a continuous assay. As indicated in Figure 5, right, a linear decrease of the oxygen concentration was observed over a period of ∼30 s. The employed assay, revealed a specific O_2_ consumption of 1506 ± 17 nmol min^−1^ mg^−1^, which is in good agreement with the specific activity of the l-carnitine depletion assay (Table 1). Accordingly, the established O_2_ depletion assay is a good alternative for the determination of carnitine monooxygenase activity.

Control assays in the absence of the reductase YeaX did not show the consumption of O_2_. However, O_2_ depletion activity was determined for experiments in which YeaW was omitted (∼10% when related to the standard assay) (Figure 5, right). The artificial YeaX catalysis resulted in the partial formation of hydrogen peroxide as determined by the ferrithiocyanate method. In agreement with this `unproductive` O_2_ consumption, previous control experiments in the absence of the catalytic unit clearly indicated the consumption of NADH [20,21]. Evidently, the sole reductase YeaX facilitates the NADH-dependent conversion of O_2_. Oxidation of NADH without TMA formation has been already described for a variant carnitine monooxygenase [17], which might be a result of an uncoupling that prevents the delivery of reducing equivalents to the active site of the enzyme.

Considering these results, the O_2_ depletion assay, but also the previously described NADH depletion assay must be applied with caution. For example, in the context of inhibition experiments, uncoupling of the carnitine monooxygenase electron transfer chain might still be associated with a linear decrease of the O_2_ or NADH concentration. However, combined information from differing types of assays might lead to a deeper understanding of, e.g., inhibition experiments.

### Carnitine monooxygenase activity under low oxygen conditions

The human intestinal tract encompasses a steep oxygen gradient. It was demonstrated that the oxygen concentration drops along the radial axis from the intestinal submucosa to the lumen. Different experimental approaches revealed values for the oxygen partial pressure ranging from ∼6% (42 mm Hg) down to ∼0.4% (3 mm Hg) [31]. Therefore, l-carnitine and the O_2_ depletion assays were also performed under hypoxic conditions (Supplementary Figure S2). In the presence of ∼5% or ∼0.4% oxygen, specific activities of 160 ± 48 or 120 ± 2 nmol min^−1^ mg^−1^ were observed in the l-carnitine depletion assay. The related O_2_ depletion experiments revealed activities of 196 ± 7 or 81 ± 3 nmol min^−1^ mg^−1^, respectively. These findings clearly indicate efficient YeaX/YeaW catalysis under low oxygen conditions. Consistent with this, enzymatic activity for the orthologous YeaW protein from *Providencia rettgeri* in the presence of 0.2% oxygen has been demonstrated [22]. It was concluded that hypoxic conditions of the human gut facilitate for the YeaX/YeaW-dependent formation of TMA.

### Substrate-related inhibition experiments

**Figure 6:**
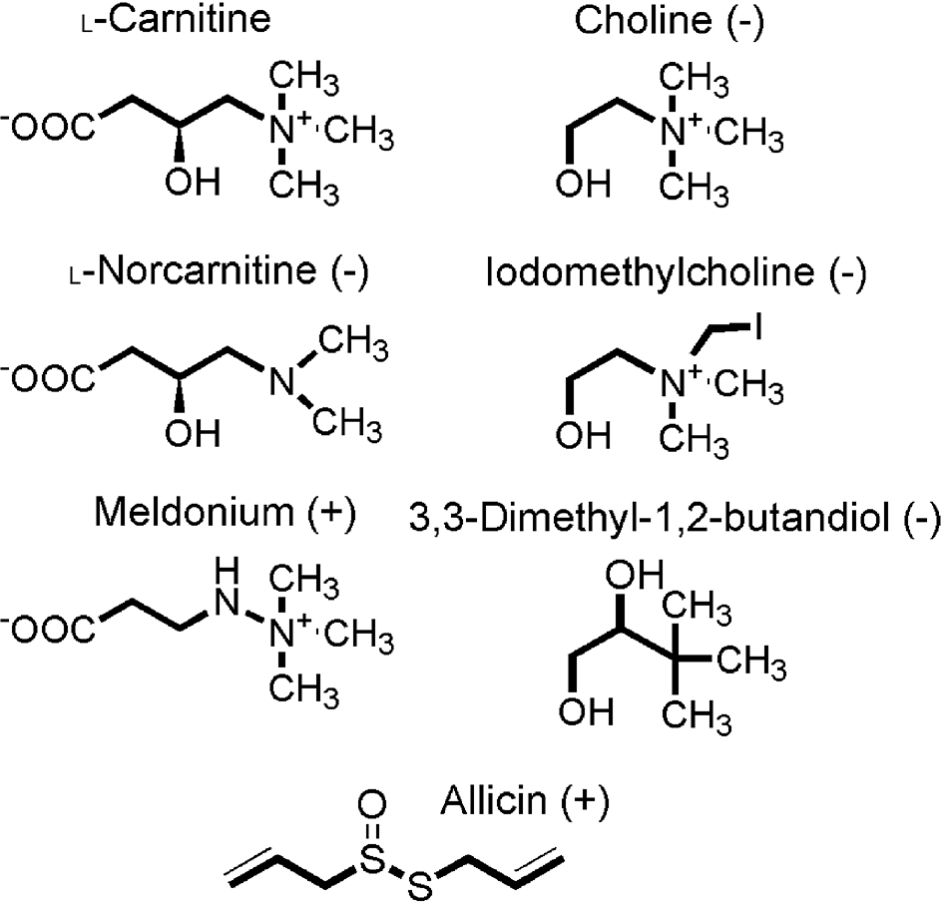
Compounds tested as potential inhibitors of carnitine monooxygenase Kinetic inactivation of the YeaX/YeaW system was explored. Inhibitors are indicated (+).

Weak enzymatic activity of different carnitine monooxygenase enzymes towards choline has been demonstrated [22]. Accordingly, structural analogs of l-carnitine and choline were investigated for the inhibition of YeaX/YeaW. Figure 6 summarizes the employed compounds. Choline-related analogs 3,3-dimethyl-1,2-butandiol or iodomethyl choline and l-carnitine derived compound L-norcarnitine did not reveal detectable carnitine monooxygenase inactivation.

### Inhibition of carnitine monooxygenase in the presence of meldonium

Suppression of microbiota-dependent production of TMAO in the presence of meldonium has been demonstrated in the animal model [32]. Therefore, potential inhibition of carnitine monooxygenase on the basis of this cardioprotective drug was investigated. Meldonium concentrations ranging from 100 to 1000 µM were analyzed in the optimized l-carnitine depletion assay (Table 1). A half-maximal inhibitory concentration (IC_50_) of 612 ± 68 µM was determined.

Identical inhibition experiments, analyzed by the O_2_ depletion assay revealed a completely different picture. Increasing meldonium concentrations in the range of 100–1000 µM resulted in an O_2_ consumption of 107–120% when related to the standard assay. These findings clearly indicate that the consumption of O_2_ does not reflect the enzymatic conversion of l-carnitine. Presence of meldonium might lead to an uncoupling of O_2_ depletion from the productive conversion of l-carnitine. Accordingly, the sole O_2_ depletion assay is not appropriate for the investigation of meldonium inhibition experiments.

On the basis of a spectrometric NADH depletion assay, meldonium has been recently described as an artificial substrate of the carnitine monooxygenase from *A. baumannii* showing a Vmax of ∼74% when related to the l-carnitine substrate [20]. Accordingly, the YeaX/YeaW-dependent conversion of meldonium (in the absence of l-carnitine) was investigated. Quantitative gas GC-MS was used to detect the possible formation of TMA with maximum sensitivity. A specific activity of ∼68 nmol min^−1^ mg^−1^ was determined. This corresponds to an activity of 3.8% when related to the natural substrate l-carnitine.

In summary, moderate carnitine monooxygenase inhibition in the presence of meldonium was demonstrated, which is paralleled by a strong uncoupling effect.

### Inhibition of carnitine monooxygenase in the presence of allicin

The organosulfur compound allicin (Figure 6) is mainly found in garlic which has long been associated with health benefits [33]. In the mouse model, dietary allicin reduces the conversion of l-carnitine to TMAO through impact on gut microbiota [34].

As an initial experiment, a freshly prepared garlic extract was used in an *in vitro* carnitine monooxygenase experiment. The l-carnitine depletion assay indicated a substantially reduced enzyme activity of 399 or 144 nmol min^−1^ mg^−1^ when the reaction mixture contained an estimated concentration of ∼200 µM or ∼2 mM allicin.

Subsequently, the l-carnitine depletion assay and the O_2_ depletion assay was performed in the presence of varying concentrations (10–100 µM) of chemically synthesized allicin [35]. As indicated in Table 1, decreasing residual activities of 93–17% were obtained. From these values, a half maximal inhibitory concentration (IC_50_) of 61 ± 3 µM was calculated (∼8 min incubation). However, inhibition efficiency was clearly dependent on pre-incubation time. Almost linear decrease of the residual activity with increasing incubation time was observed (compare Table 1) for both types of depletion assays. Allicin is described as a reactive thiol-trapping sulfur compound [36]. Time-dependent covalent modification of carnitine monooxygenase might be responsible for the observed enzyme inactivation.

### Toward the protein–protein interaction of YeaX and YeaW

YeaX comprises a tightly bound FMN cofactor, which implies direct protein–protein interaction of YeaX with the catalytic component YeaW to facilitate intercomponent electron transfer. However, carnitine monooxygenase complex formation has not been exemplified to date. Our attempts to ‘trap’ the YeaX/YeaW interaction, made use of high concentrations of the substrates l-carnitine and NADH. Experiments were performed in the absence of molecular oxygen to prevent reaction product formation. However, protein–protein interaction experiments using immobilized YeaX (or YeaW) did not allow for the trapping of YeaW (or YeaX). Therefore, transient (or weak) interaction of monomeric YeaX and trimeric YeaW was concluded.

### Evolutionary conservation of the docking interface: reconstitution of chimeric carnitine monooxygenases activity

Intercomponent electron transfer is often facilitated via evolutionarily conserved protein docking faces [37,38]. Accordingly, chimeric carnitine monooxygenase enzymes consisting of individual subunits from *E. coli* (YeaX or YeaW) and *A. baumannii* (CntA or CntB) were reconstituted under conditions of the optimized assay. The specific activity of the homologous *E. coli* system was set as 100% and all other activities were related to that. The kinetic experiment for YeaX in combination with CntA and the assay for CntB in combination with YeaW revealed enzymatic activities of 100 ± 4 and 20 ± 2%, respectively (Table 1). Based on these findings, it was concluded that the docking surfaces responsible for protein–protein interaction and electron transfer were partially conserved during the evolution of carnitine monooxygenase.

### Structural modeling of YeaX and YeaW

**Figure 7:**
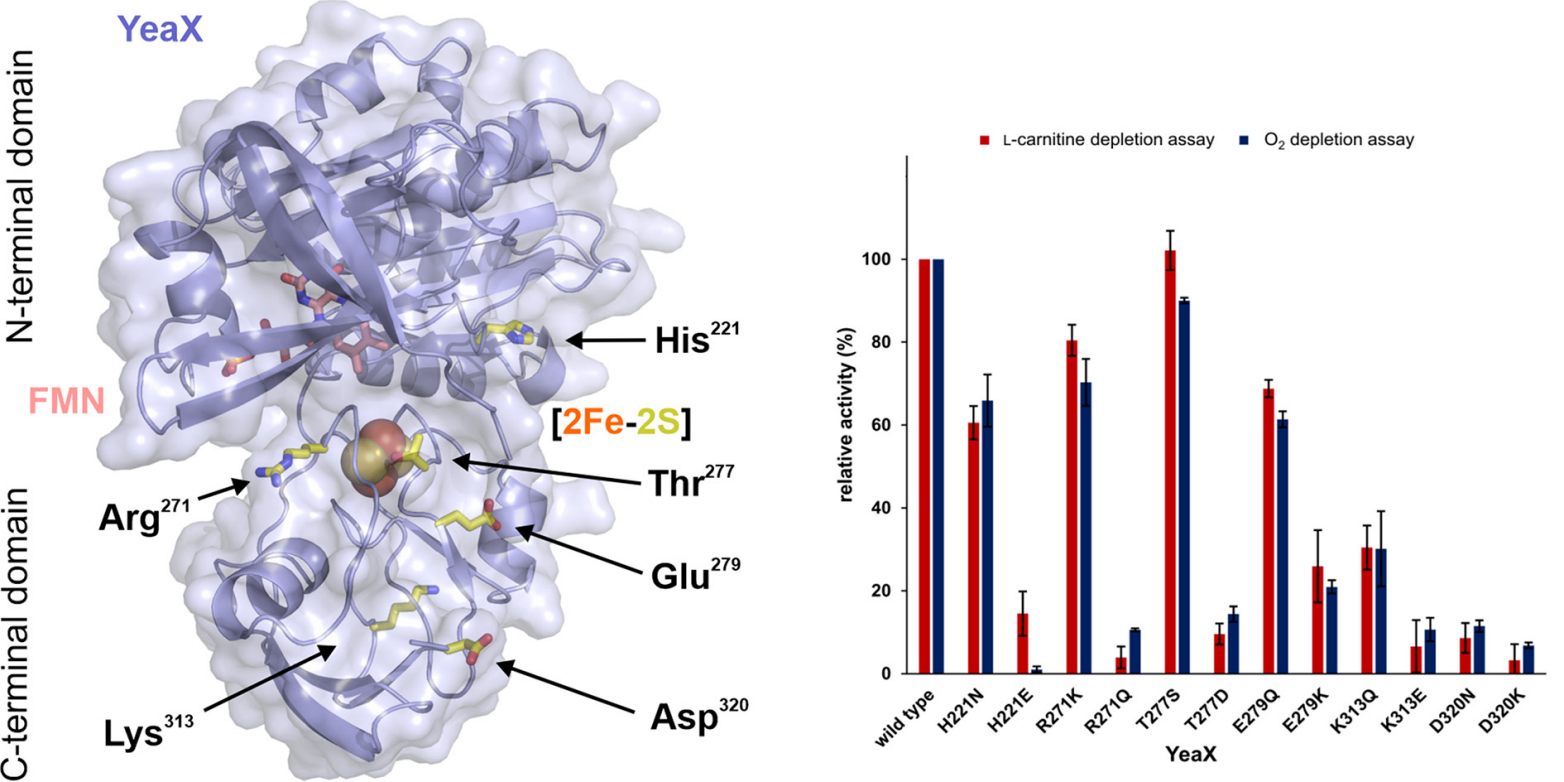
Structural modeling of YeaX and enzymatic activity of site-directed mutants of YeaX The AlphaFold [39] model (left) comprises the N-terminal domain (top, residues 1–126) harboring the FMN cofactor (pale pink). The C-terminal domain (bottom, residues 127–318) contains the [2Fe-2S] cluster (spheres). Redox cofactors were superimposed according to structures with PDB IDs: 6LAA and 2PIA. Highly conserved YeaX residues were identified (yellow sticks) and subsequently explored in a structure guided mutagenesis approach. The figure was created with PyMOL [57]. Enzymatic activity of site-directed mutants of YeaX (right). Purified recombinant proteins were subjected to the l-carnitine depletion assay (red) and the O_2_ depletion assay (blue). The integrity of all proteins was confirmed by thermal denaturation experiments and UV-visible absorption spectroscopy. Specific activities in the presence of the wild-type protein were set as 100%, and all kinetic data were related to these values.

**Figure 8:**
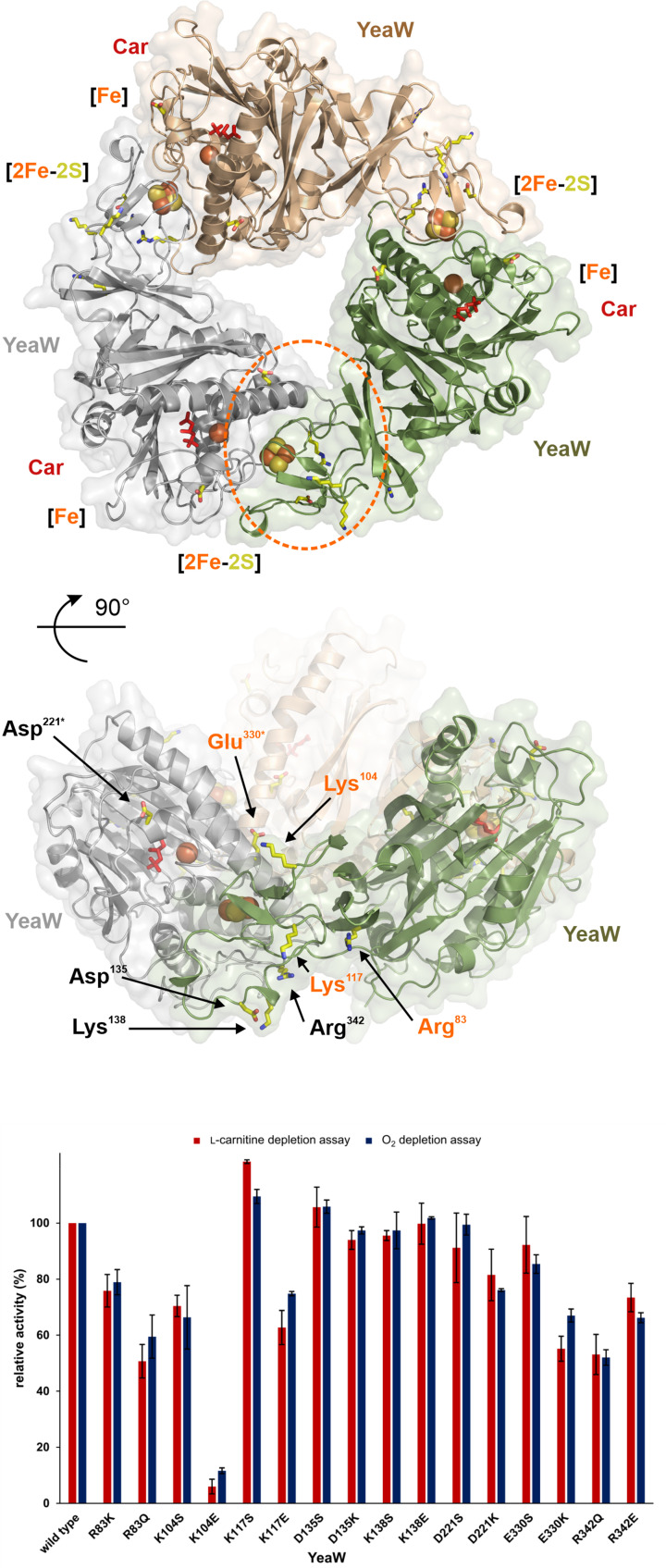
Structural modeling of the YeaW trimer and enzymatic activity of site-directed mutants of YeaW AlphaFold [39] was used to model the structure of the YeaW monomer, which was superimposed to the experimental structure of trimeric CntA (PDB ID: 6Y9D, root-mean-square deviation: 0.46 Å). The resulting (YeaW)_3_ structure (top view, upper panel; front view, middle) exists as a head-to-tail trimer that enables the electron transfer between the Rieske-type [2Fe-2S] cluster (brown and yellow spheres) and the [Fe] (brown sphere) of adjacent YeaW subunits (coloured green, gray and brown). The substrate l-carnitine (Car) is indicated in red. Highly conserved YeaW residues were identified (yellow sticks) and investigated in a structure guided mutagenesis study. The proposed docking face for the protein–protein interaction with YeaX and the related electron transfer is highlighted (upper panel, dashed orange oval). YeaW variants with exchanged residues Arg^83^, Lys^104^, Lys^117^ or Glu^330*^ (middle, highlighted orange) showed only minor residual enzymatic activities. The figure was created with *PyMOL* [57]. Enzymatic activity of site-directed mutants of YeaW (lower panel). Purified recombinant proteins were subjected to the l-carnitine depletion assay (red) and the O_2_ depletion assay (blue). The integrity of all proteins was confirmed by thermal denaturation experiments and UV-visible absorption spectroscopy. Specific activities in the presence of the wild type protein were set as 100% and all kinetic data were related to these values.

AlphaFold is a new machine learning approach that allows for highly accurate protein structure prediction [39]. The resulting 3D models of YeaX and YeaW are depicted in Figures 7 and 8. Redox cofactors were superimposed according to structures with protein data bank (PDB) IDs: 6LAA, 2PIA and 6Y9D. Sequence alignments of orthologous YeaX and YeaW proteins were calculated and the degree of sequence conservation was assigned to both structural models aiming to identify surface localized amino acid residues of central importance for the spatial interplay of subunit YeaX and YeaW and/or the related electron transfer processes. Based on these theoretical experiments, a structure guided mutagenesis approach comprising 12 YeaX and 16 YeaW variant proteins was performed. Individual amino acid positions were replaced by a conservative amino acid exchange (e.g., similar size and polarity) and subsequently by a more drastic amino acid alteration (e.g., inducing charge reversal). The integrity of the purified protein mutants was confirmed by thermal denaturation experiments and UV-visible absorption spectroscopy (Supplementary Figure S3). The specific activity of all proteins was determined using the l-carnitine depletion assay and the O_2_ depletion assay (substrate depletion assays). Furthermore, the ‘uncoupled’ O_2_ consumption of all YeaX variants in the absence of the catalytic component YeaW was determined (uncoupled assay). The specific activities in the presence of wild-type protein(s) were set as 100% and all kinetic data were related to these values (Figures 7 and 8).

### Modeling and mutagenesis of YeaX

The AlphaFold model of YeaX (Figure 7) comprises a non-globular tertiary structure as also indicated by the results of the size exclusion chromatography experiments. It includes an N-terminal domain (top, residues 1–126) which harbors the NADH and FMN binding site (bound FMN in pale pink). As also indicated by a recent investigation [21], residues Glu^77^ and Ser^84^ of YeaX might contribute to the tight binding of the FMN cofactor (not depicted). The C-terminal domain (bottom, residues 127-318) facilitates for the binding of the [2Fe-2S] cluster via ligands Cys^270^, Cys^275^, Cys^278^ and Cys^308^ (not depicted) in agreement with the results of a recent *A. baumannii* mutagenesis study [21]. A DALI search revealed the following structural homologs in the protein data bank: phthalate dioxygenase reductase (PDB ID: 2PIA) for the N-terminal domain and self-sufficient P450 monooxygenase (PDB ID: 6LAA) for the C-terminal part of YeaX.

Functional relevance of residue His^221^ of the N-terminal domain was demonstrated by mutagenesis. Variant H221N revealed residual l-carnitine depletion (or O_2_ depletion, from now on in brackets) activity of 61% (66%). Stronger inactivation was determined for mutant H221E with 15% (2%) activity.

In the absence of the catalytic component YeaW, even stronger reduction of the artificial O_2_ consumption with 21% for H221N and 0% for H221E was measured (O_2_ consumption without YeaW set as 100%). In the present study, solely mutation of residue His^221^ resulted in a more pronounced enzyme inactivation in the uncoupled assay (without YeaW) when compared with the substrate depletion assay(s). It was hypothesized that mutations H221N and H221E impact the NADH-dependent electron transfer steps of YeaX, which also hampers significantly the artificial reduction of O_2_ in the absence of YeaW. Impairment of the stacking interaction of His^221^ with the conserved residue Tyr^195^ and/or the polar contact with His^193^ might affect the initial electron transfer steps of YeaX.

Amino acid Arg^271^ is located directly next to cluster ligand Cys^270^ with an outward facing guanidino group. Variant R271K showed residual l-carnitine depletion (or O_2_ depletion) activity of 80% (70%), whereas mutant R271Q only revealed residual activity of 4% (11%). Obviously, a positively charged side chain is essential to maintain enzymatic activity. Salt bridge formation between Arg^271^ and Glu^259^ might be important for the appropriate positioning of the redox-active [2Fe-2S] cluster of YeaX. Hypothetical interaction of Arg^271^ with the catalytic unit YeaW was considered.

Thr^277^ is located on the protein surface in the cluster binding loop region between ligands Cys^275^ and Cys^278^. In variant T277S, a serine residue can act as a perfect substitute indicated by enzymatic activity of 102% (90%). By contrast, the sterically more demanding exchange T277D shows strong impact delineated by a residual activity of only 10% (14%). The aspartate residue might have an influence on the electron transfer and/or the spatial orientation of the [2Fe-2S] cluster of YeaX.

Mutagenizing the outward facing residue Glu^279^ indicated moderate loss of enzymatic function for variant E279Q with an activity of 69% (61%). This ‘isosteric’ exchange did not indicate reduced O_2_ consumption in the uncoupled assay. However, introducing the positively charged side chain in mutant E279K revealed a differing activity profile. Substrate depletion activity of 26% (21%) was determined, paralleled by a substantial loss of the uncoupled activity with a relative value of 23%. Involvement of Glu^279^ in the [2Fe2S]-mediated electron transfer on to YeaW was proposed.

In the modeled structure, the positively charged side chain of Lys^313^ is oriented toward the protein surface. Variant K313Q indicated residual activity of 30% (30%). Charge reversal of mutant K313E resulted in a more pronounced activity reduction with 7% (11%) residual activity. These findings are in agreement with a central role of Lys^313^. Considering the large distance of Lys^313^ to the [2Fe-2S] cluster of YeaX, a direct interaction of this residue with the catalytic unit YeaW was inferred.

Surface exposed residue Asp^320^ is closely located to the C-terminal end of YeaX. In the present model Asp^320^ forms a bidentate salt bridge to Arg^241^. Relevance of this interaction might be indicated by an activity of 9% (11%) for the conservative mutation D320N and also by a residual activity of only 3% (7%) for variant D320K. Asp^320^ is adjacent to a hydrophobic surface patch showing a high degree of sequence conservation (Ile^282^, Leu^321^). Accordingly, this part of the YeaX molecule might be relevant for the protein–protein interaction of YeaX and YeaW.

### Modeling and mutagenesis of YeaW

The monomeric AlphaFold model of YeaW was superimposed to the experimental structure of trimeric CntA (PDB ID: 6Y9D, root-mean-square deviation of 0.46 Å), which resulted in an accurate oligomer assembly with no steric clashes between the symmetry-related protomers (Figure 8). The resulting (YeaW)_3_ model exists as a head-to-tail trimer that enables for the electron transfer between the Rieske-type [2Fe-2S] cluster and the [Fe] of adjacent YeaW subunits (colored green, gray and brown). In agreement with recent investigations, residues Cys^89^, His^91^, Cys^109^ and His^112^ (not depicted) allow for the binding of the Rieske-type [2Fe-2S] cluster. Furthermore, side chains of His^211^, His^216^ and Asp^325^ can function as ligands of the mononuclear [Fe] which is located in close proximity to the substrate l-carnitine (red) [20,21].

In Figure 8, mutagenized residues are shown on adjacent subunits of YeaW (Asp^221*^, Glu^330*^ and Lys^104^, Asp^135^, Lys^138^, Arg^342^, Lys^117^, Arg^83^; residues from adjacent protomers indicated by ^*^). The highlighted portion (dashed orange oval) of the ring shaped protein might provide one of the symmetry related docking surfaces, which facilitate for the electron transfer from YeaX.

Functional relevance of the mutated residues was accessed in the l-carnitine depletion (or the O_2_ depletion assay, values in brackets). Among these conserved residues, the following variants only revealed a minor reduction of the activity: D221S 91% (99%), D221K 81% (76%), D135S 106% (106%), D135K 94% (97%), K138S 96% (97%) and K138E 100% (102%). These findings clearly rule out a central role of Asp^221*^, Asp^135^ and Lys^138^ for the electron transfer or the protein-protein interaction of carnitine monooxygenase.

Functional relevance of Arg^342^ was indicated since variant R342Q revealed a residual activity of 53% (52%) and R342E of 73% (66%). In the structural model, the downward facing side chain of Arg^342^ (Figure 8) is engaged in polar contacts with the main chain of the adjacent YeaW subunit. Accordingly, this residue of the trimer interface might have an indirect influence on the inter-subunit electron transfer within (YeaW)_3_. Residues Glu^330*^, Lys^104^, Lys^117^ and Arg^83^ delineate the central cavity that is located between neighboring monomers on the upper side of (YeaW)_3_ (Figure 8). Conservative mutations of these residues revealed only minor (or no) inactivation of YeaW: E330S 92% (85%), K104S 70% (66%), R83K 76% (79%) and K117S 122% (109%). Charge alteration of these surface localized amino acid residues resulted in a substantial decrease of the enzyme activity: E330K 55% (67%), K104E 6% (12%), R83Q 51% (59%) and K117E 63% (75%). From these findings it was hypothesized that Glu^330*^, Lys^104^, Lys^117^ and Arg^83^ play a central role for the protein–protein interaction and/or electron transfer processes of carnitine monooxygenase. In accordance with these data, the C-terminal domain of YeaX would approach the Rieske-type cluster of YeaW from above, and subsequently binds to the deepening of two adjacent YeaW subunits (compare Figure 8).

## Discussion

TMAO is a gut microbiota associated risk factor which promotes atherosclerosis and the progression of cardiovascular disease. Dietary l-carnitine is metabolized by the gut microbial enzyme system YeaX/YeaW. The resulting reaction product TMA enters the host circulation and is further converted to TMAO in the liver [40,41]. Inhibition of gut microbial TMA production is a promising strategy for cardiovascular disease prevention [16,20,42]. In this context, the molecular understanding of the related enzyme catalysis might pave the way for the development of new inhibition strategies.

*In vitro* investigation of the established YeaX/YeaW system required the optimization of enzymatic reaction conditions. Increased activity in response to the addition of Fe^2+^ was observed in agreement with related investigations. Rieske-type enzymes solely provide two histidine and one aspartate (or glutamate) ligand for the binding of the mononuclear [Fe] center, whereas weakly bound water completes the coordination sphere. It is well described that an incomplete occupancy of the [Fe] center can be compensated by external addition of Fe^2+^ [43–45]. Partial uncoupling of NADH and O_2_ consumption from reaction product formation can lead to the emergence of reactive oxygen species. Subsequently, these molecules can cause irreversible enzyme inactivation [30]. Performing YeaX/YeaW reactions in the presence of catalase might lead to a reduction of such inhibiting effects. Overall, the optimized activity assay in the presence of Fe^2+^ and catalase revealed a 1.7-fold increase in the carnitine monooxygenase activity.

The presented characterization of the redox active components of the *E. coli* YeaX/YeaW system is in good agreement with the recently proposed redox catalytic cycle of *A. baumannii* CntB/CntA [21] (Figure 2). YeaX acts as a monomeric reductase harboring an FMN cofactor and a plant-type [2Fe-2S] cluster, which is ligated by residues Cys^270^, Cys^275^, Cys^278^ and Cys^308^. This allows for the electron delivery from NADH via FMN on to the [2Fe-2S] cluster of YeaX. Reduced YeaX enables for the electron transfer on to trimeric YeaW. YeaW carries a Rieske-type [2Fe-2S] cluster ligated by residues Cys^89^, His^91^, Cys^109^ and His^112^. Side chains of His^211^, His^216^ and Asp^325^ were identified as ligands of a mononuclear [Fe] center, facilitating reductive activation of molecular oxygen. This step is triggered by electron transfer from YeaX onto the [Fe] center of YeaW via the Rieske [2Fe-2S] cluster. Redox catalysis of trimeric YeaW includes the electron transfer between neighboring protomers (compare Figure 2). This is strongly supported by the calculated AlphaFold model which was subsequently assembled as a trimer using coordinates of an orthologous X-ray protein template. The (YeaW)_3_ model conclusively indicated complementarity at the protomer–protomer interface. Rieske-type oxygenases contain evolutionarily conserved contact regions that are centered by a bridging aspartate or glutamate residue [17,18]. The respective Glu^208^ of YeaW is appropriately positioned to facilitate the intermolecular electron transfer of adjacent YeaW protomers, underscoring the quality of the quaternary structural model.

At the present point, AlphaFold prediction of the overall YeaW trimer did not result in an oligomer assembly which was in agreement with any experimental Rieske-type protein structure. Accordingly, the combination of modelling results with experimental evidence will be (still) inevitable for the efficient implementation of AlphaFold predictions.

In the present study, two different molecules with proposed cardioprotective properties were exemplified as carnitine monooxygenase inhibitors. Meldonium is a pharmaceutical substance that is approved in Eastern Europe for cerebral and myocardial ischemia [46]. Besides this, suppression of microbiota-dependent production of TMAO in the presence of meldonium has been demonstrated in a rat model [32]. Meldonium inhibition experiments revealed an IC_50_ of 612 µM, which was paralleled by a pronounced uncoupling of the reaction. Under *in vivo* conditions, inhibition strategies that cause uncoupling of oxygen activation and substrate conversion might be of special interest. Meldonium might not only inhibit the formation of TMA, but it might also damage the catabolism of l-carnitine as a result of reactive oxygen species.

The present study also demonstrated the slow conversion of meldonium in the presence of YeaX/YeaW. Thus, the pharmaceutical application of meldonium should also take into account the microbiome-dependent metabolism in the human gut.

Overall, our meldonium inhibition experiments indicated a very characteristic ‘activity profile’, which is clearly related to other Rieske-type oxygenases. As an example, benzene-induced uncoupling of naphthalene dioxygenase is well described in the literature. Presence of the artificial substrate benzene resulted in the formation of reactive oxygen species with simultaneous inactivation of the dioxygenase activity [30].

Allicin has long been associated with many health benefits, which also include a positive influence on the cardiovascular system [33]. Furthermore, reduced conversion of l-carnitine to TMAO through impact on gut microbiota has been described [34]. Under conditions of the present investigation, an IC_50_ of 61 µM was determined and a clearly time-dependent inhibitory effect was demonstrated. Increasing inhibition potency with time is an indicator for irreversible enzyme inactivation [47]. Allicin reacts with free sulfhydryl groups resulting in *S*-allylmercapto modification [36]. Accordingly, covalent *S*-allylmercapto cysteine formation might be related to the inactivation of carnitine monooxygenase. The sequences of YeaX and YeaW reveal an overall of six and seven cysteine residues, which are not involved in [Fe-S] cluster ligation. Future experiments should be focused on the further understanding of the inactivation of YeaX or YeaW in the presence of allicin. In this context, potential ‘attacking’ of redox active [Fe-S] clusters must be taken into account as a promising research perspective which has not been addressed in the literature so far.

The diverse family of Rieske-type oxygenases typically comprise a specific reductase, eventually a ferredoxin and the terminal oxygenase component. YeaW was recently ascribed to the novel group V of two-component Rieske-type oxygenases, which enable the degradation of quaternary ammonium compounds [48,49]. Although the structure of many Rieske-type oxygenases and of the respective redox partners are known, there is no available structural information on a reductase/oxygenase complex. Thus, there is a gap in our understanding on the two-component interaction and the involved electron transfer processes. On the basis of our chimeric carnitine monooxygenase assays, it was demonstrated that the orthologous systems YeaX/YeaW and CntB/CntA share a partially conserved docking interface. The YeaX/CntA experiment resulted in 100% activity whereas the combination CntB/YeaW only revealed 20% residual activity. This might indicate subtle differences between the docking surfaces of CntB and YeaX.

Based on sequence alignments and AlpaFold predictions a comprehensive structure guided mutagenesis approach was performed. Our strategy enabled the identification of individual amino acid residues with relevance for the interplay and/or electron transfer between YeaX and YeaW.

Kinetic analysis of 28 purified mutant proteins clearly indicated functional relevance of residues His^221^, Arg^271^, Thr^277^, Glu^279^, Lys^313^ and Asp^320^ of YeaX. It was hypothesized that His^221^ is involved in the early NADPH-dependent electron transfer steps of YeaX. Residues Thr^277^ and Glu^279^ were suggested to participate in the subsequent [2Fe2S]-mediated catalysis of the reductase. For Arg^271^, Lys^313^ and Asp^320^ of YeaX, a central role for the catalysis and/or the protein–protein interaction of carnitine monooxygenase was concluded. With regard to the catalytic unit (YeaW)_3_, a docking surface centered around residues Arg^83^, Lys^104^ and Lys^117^ might be concluded on the basis of the employed experimental approach. These residues delineate the central cavity that is located between neighboring monomers on the upper side of (YeaW)_3_ (compare Figures 7 and 8).

The present investigation revealed an initial picture of the docking interface between the reductase YeaX and (YeaW)_3_. Future experiments in our laboratory will focus on the crystallization of the binary protein complex to gain a molecular understanding for the triggering of the individual electron transfer steps of this fascinating enzyme.

## Experimental procedures

### Production and purification of YeaX and YeaW

Genes for the production of YeaX or YeaW from the gut microbial strain *E. coli* KO11FL [25] were PCR amplified from *E. coli* DH10B (both strains share 100% identical YeaX and YeaW sequences, respectively). Primers GGGTACCAGGATCCAATGTCAGACTATCAAATGTTTG and CTGCGGCCGCGTCGACCTACAAATCCAACACCAGG or CCGGGTACCAGGATCCAATGAGCAATCTGAGCCCTGAC and CTGCGGCCGCGTCGACTTAGTCCTTAAACACCTGCGCC were used. Amplicons were inserted into the BamHI/SalI restriction sites of vector pET52b(+) to yield plasmids pET52b(+)-*yeaX* and pET52b(+)-*yeaW*, respectively. Recombinant YeaX or YeaW fusion proteins with an N-terminal strep tag II were overproduced in *E. coli* Tuner (DE3) cells. Respective overnight cultures were used to inoculate 4× 500 ml (pET52b(+)-*yeaX*) or 500 ml (pET52b(+)-*yeaW*) LB medium in baffled flasks containing 100 µg ml^−1^ ampicillin. After ∼2 h at 37°C and 200 rpm, an optical density at 578 nm of 0.5–0.6 was reached. The production of YeaX or YeaW was initiated by addition of 50 µM isopropyl-β-D-thiogalactopyranoside (IPTG), 1 mM Fe(III)-citrate and 1 mM L-cysteine hydrochloride (to improve iron center maturation), respectively. After 16 h of cultivation at 17°C and 180 rpm, cells were harvested by centrifugation at 4000 × *g* for 15 min at 4°C, resuspended in 100 mM 4-(2-hydroxyethyl)-1 piperazine ethanesulfonic acid (HEPES)-NaOH pH 7.5, 150 mM NaCl, 10 mM MgCl (buffer 1). Cells were sedimented and stored at −20°C until further use. The pellet from 4× 500 ml or 500 ml cell culture was thawed and resuspended in 40 or 10 ml buffer 1 containing one tablet protease inhibitor mix (Complete Mini, EDTA-free Protease Inhibitor Cocktail, Roche) and 2.5 units/ml Turbo Nuclease (Jena Bioscience). Cells were disrupted by a single passage through a French press at 14,500 psi, respectively. Lysates were clarified at 112000 × *g* for 65 min at 4°C. Supernatants were supplemented with avidin (0.6 U/ml), passed through a 0.45 µm filter and subsequently applied to a column containing 1.5 ml Strep-Tactin Superflow high capacity resin (IBA Lifesciences). Affinity columns were washed with 2× 1.5 ml buffer 1 to remove unbound proteins. YeaX or YeaW was eluted with 4× 1.5 ml buffer 1 containing 5 mM desthiobiotin. Target protein fractions were identified by SDS-PAGE and subsequently shock frozen in liquid nitrogen. If required, purified protein samples were concentrated using Amicon Ultra-0.5 Centrifugal Filter Units (Millipore) equipped with a 30000 Da cut-off membrane or dialyzed against e.g. buffer 1.

### Protein concentrations

The concentration of purified YeaX and YeaW was determined using Bradford reagent (Sigma-Aldrich) according to the manufacturer’s instructions with bovine serum albumin as a standard.

### Analytical size exclusion chromatography

The native molecular mass of YeaX and YeaW was analyzed by size exclusion chromatography using a Superdex 200 increase 5/150 GL column (GE Healthcare). Protein standards (molecular weight marker kit MWGF1000, Sigma) in the presence of 10 mM Hepes-NaOH, pH 7.5, 150 mM NaCl at a flow rate of 0.45 ml min^−1^ were used for calibration. Samples of 20 µl (∼300 µM) were injected and elution was monitored at 280 and 445 nm.

### Absorption and fluorescence spectroscopy

Purified samples of YeaX and YeaW were subjected to UV-visible light absorption spectroscopy using a V-650 spectrophotometer (Jasco). The FMN content of purified YeaX was quantified by absorption spectroscopy using an extinction coefficient of ϵ_450_ = 12.2 mM^−1^cm^−1^ for free FMN. FMN was liberated by heat denaturation. A purified sample of YeaX (∼50 µM) in a volume of 300 µl was incubated at 99°C for 10 min. The liberated cofactor was separated from the denatured protein by centrifugation (16000 × *g*, 10 min at 22°C).

Fluorescence spectra were recorded on a FP-8500 spectrofluorometer (Jasco). The pH-dependent fluorescence of equimolar FAD and FMN standards was used to initially identify the flavin cofactor of YeaX as described in [27].

### Cofactor determination by high-performance liquid chromatography (HPLC)

The supernatant after denaturation of YeaX or YeaW (see above) was analyzed on a Reprosil 100 C18 column (Techlab) as detailed in [21].

### Iron determination method

Protein bound iron was determined colorimetrically with o-phenanthroline after acid denaturation of purified YeaX or YeaW [50].

### EPR spectroscopy

Purified samples of YeaX and YeaW were concentrated (∼500 µM) and sodium dithionite (2, 5 or 10 mM) was added as indicated. After an incubation time of 10 min, samples were transferred into 3 mm EPR tubes and frozen in liquid nitrogen. A Bruker Elexsys E-500 CW X-band spectrometer was used to record X-Band EPR spectra. Samples were placed in a standard TE102 resonator and low temperature measurements were conducted using an Oxford ESR 900 helium flow cryostat (3–300 K). Baseline corrections were performed by subtracting a background spectrum, obtained under the same experimental conditions from an empty tube.

### Optimization of the l-carnitine depletion assay

For the investigation of the YeaX/YeaW system, the recently described l-carnitine depletion assay [21] has been optimized. A typical set of three interleaved activity experiments was based on 1.5 ml buffer 1 containing 15 µM YeaX, 5 µM YeaW and 300 µM l-carnitine. From this master mix, 385 µl were combined with 40 µl of a 20 mM NADH solution to initiate enzyme catalysis, respectively. At defined time points (30, 60, 90, 120 and 300 s), samples of 25 µl were taken and subsequently heat inactivated by the addition of 50 µl buffer 1 which was pre-incubated at 99°C. Samples were further incubated for 10 min at 99°C, stored on ice for 5 min and subsequently centrifuged for 10 min at 12000 × *g*. Supernatants were centrifuged again and the l-carnitine concentration from 25 µl of the respective supernatants (or from the master mix) was determined as detailed recently. Under dimmed light conditions, the coupled reaction of carnitine acetyltransferase in the presence of acetyl-coenzyme A was followed spectroscopically due to the cleavage of 5,5′-dithiobis-(2-nitrobenzoic acid) (DTNB) [21].

Optimization of the l-carnitine depletion assay in the presence of YeaX/YeaW was performed at temperatures ranging from 17 to 37°C. Furthermore, the influence of 9–90 µM FeSO_4_ and the addition of 360–2200 nM catalase [30] was investigated.

### O_2_ depletion assay

A fiber-optic oxygen meter (FireSting) fitted with a spot-fiber oxygen sensor (OXR50) from PyroScience was used for the direct measurement of the carnitine monooxygenase-dependent O_2_ consumption. About 160 µl of the air-saturated reaction mixture of the optimized l-carnitine depletion assay was measured over a period of 2 min at 27°C. The specific O_2_ depletion activity was determined in the linear range of the assay. Control reactions in the absence of YeaX, YeaW or in the absence of FeSO_4_ and catalase were performed. The formation of hydrogen peroxide was monitored by the ferrithiocyanate method as detailed elsewhere [51].

### Activity measurements under low oxygen conditions

The l-carnitine depletion assay and the O_2_ depletion assay were also performed in an anaerobic chamber (Coy Laboratories) using N_2_ saturated buffers. Appropriate mixtures of oxygen-free and air saturated assay components facilitated for the adjustment of a specific oxygen partial pressure. A fiber-optic oxygen meter (FireSting, PyroScience) was used to validate the resulting oxygen values.

### Inhibition of the YeaX/YeaW system

The l-carnitine depletion assay and the O_2_ depletion assay (also in the absence of YeaW) were carried out in the presence of 3,3-dimethyl-1,2-butandiol (Sigma-Aldrich), iodomethyl choline (ABCR-Chemicals), L-norcarnitine (Toronto Research Chemicals), meldonium (Sigma-Aldrich) and allicin. The latter compound was obtained by chemical synthesis as detailed in [35]. Alternatively, a freshly prepared garlic extract was added to activity experiments. The extract was prepared by grinding 21 g fresh garlic with 15 ml buffer 1, followed by two centrifugation steps at 12000 × *g* (10 min, 4°C), respectively [52]. The amount of bioavailable allicin in the resulting supernatant was roughly estimated taking into account an average content of 3.6 mg allicin per g of fresh garlic [53]. In all cases, control reactions in the absence of YeaX and/or YeaW were performed. Experiments were performed in triplicate and (at least) three independent experiments were conducted.

### TMA quantification by GC-MS

Potential enzymatic conversion of the substrate-related compound meldonium was analyzed by GC-MS. Therefore, the natural substrate of carnitine monooxygenase was replaced by 300 µM meldonium in the optimized l-carnitine depletion assay. Samples of 425 µl were inactivated at time points 30 s, 1, 2 and 5 min. Reactions were stopped by the addition of 425 µl 1 M HCl. TMA formation was quantified (GANZIMMUN Diagnostics AG), according to established procedures [54], and the specific activity for the conversion of meldonium was calculated. Control experiments in the absence of NADH or in the presence of l-carnitine were processed accordingly.

### Reconstitution of chimeric carnitine monooxygenase activity

The orthologous reductase CntA and the catalytic unit CntA from *A. baumannii* were recombinantly overproduced and purified as described recently [21]. Artificial carnitine monooxygenase enzymes were reconstituted under conditions of the optimized l-carnitine depletion assay at 27°C. Individual *E. coli* subunits YeaX or YeaW were combined with the respective *A. baumannii* componenents CntB or CntA, respectively (15 µM reductase, 5 µM oxygenase). The specific activity of the homologous YeaX/YeaW system was set as 100% and all other values were related to that. Relative O_2_ depletion activities were determined accordingly. All kinetic experiments were performed in triplicate and the results were reproduced three times.

### Bioinformatic analyses

Homologous YeaX and YeaW protein sequences were identified by BLAST analyses [55], amino acid sequence alignments were generated by Clustal Omega [56]. AlphaFold [39] was used for the modeling of the 3D monomer structures of both proteins (AlphaFold entry IDs: P76254 and Q8K3I6). PyMOL was used for the superpositioning of protein structures (ALIGN command) and to visualize the resulting models [57]. Cofactors of YeaX were superimposed according to PDB IDs: 2PIA and 6LAA [58,59]. Overlay of the monomeric AlphaFold structure onto coordinates of PDB ID: 6Y9D [20] revealed the metal cofactor containing model of the YeaW trimer.

### Variant proteins of YeaX and YeaW

Variant plasmids (of pET52b(+)-*yeaX* or pET52b(+)-*yeaW*) for the production of site-directed YeaX and YeaW mutants were created using the QuikChange site directed mutagenesis kit (Agilent Technologies) using the following oligonucleotides (exchanged nucleotides bold):

R83K:CGTGATAAGGTTTTG**AAG**GCGTTCTATAACGTG, R83Q: CGTGATAAGGTTTTGC**AG**GCGTTCTATAACGTG, K104S: AGCGGTGAAGGAAAA**GC**AAGCAATGTGATTACCTGCCCGT, K104E: GAGCGGTGAAGGAAAAGCA**GAG**AATGTGATTACCTGCCCGT, K117S: CACGCATGGGCATTCA**GC**CTCGATGGCAACCTGG, K117E: TCACGCATGGGCATTC**GAG**CTCGATGGCAACCTGG, D135S: CGCCAATTTC**AG**TAGCGACAAAGCGC, D135K: GCGAAAACGTCGCCAATTTC**A**A**G**AGCGACAAAGCGCAACTG, K138S: CGTCGCCAATTTCGATA**GC**GACAGCGCGCAACTGGTT, K138E: CGATAGCGAC**G**A**G**GCGCAACTGGTTCCGG, D221S: CGCATCCAGGTTTCTCC**AG**CTCCGTACAGGTTGATC, D221K: CCAGGTTTCTCC**A**A**G**TCCGTACAGG, E330S: GATTTACGTCTGGTT**TC**AAGCGTACAGAAAGGGC, E330K: GAAGATTTACGTCTGGTT**A**AAAGCGTACAGAAAGGGC, R342E: CGTGGCTAT**GAG**GGTCAGGGGCGCATC, R320Q: AATCGCGTGGCTAT**CA**GGGTCAGGGGCGCATC (for the exchange of codons in *yeaW*) or H221N: GCCGCCGATACGCTG**A**ACTTTGAGCAATTTGC, H221E: CGCCGCCGATACGCTG**G**A**G**TTTGAGCAATTTGCTAT, R271K: CGCGAAAGTGGAATGTTTATGT**AAG**GAAGGGGTATGCGGAACCTG, R271Q: CGCGAAAGTGGAATGTTTATGTC**AG**GAAGGGGTATGCGGAAC, T277S: GTGAAGGGGTATGCGGAAG**C**TGCGAAACAGC, T277D: CGTGAAGGGGTATGCGGA**GA**CTGCGAAACAGCAATACT, E279Q: GGGTATGCGGAACCTGCCAGA**CAG**CAATACTGGAAGG, E279K: GGGTATGCGGAACCTGC**A**AAACAGCAATACTGGAA, K313Q: GTTGTTCGCGTGCGC**A**GGGTAAACGCCTG, K313E: GTTGTTCGCGTGCG**G**AGGGTAAACGCCTG, D320N: GTAAACGCCTGGTGTTG**A**ATTTGTAGGTCGACGCG, D320K: GTAAACGCCTGGTGTTG**A**A**G**TTGTAGGTCGACGCGGC (for the exchange of codons in *yeaX*). Variant proteins of YeaX or YeaW were overproduced and purified as described for the respective wild type proteins. The integrity of all proteins was analyzed by SDS-PAGE analyses and by UV-visible absorption spectroscopy. Furthermore, thermal denaturation experiments (Thermofluor assay) using a QuantStudio 1 Real-Time-PCR-System (Thermofisher Scientific) were conducted to validate the apparent melting temperature of all variant proteins. Kinetic experiments for all variant proteins were performed in triplicate as detailed for the wild-type proteins.

## Supplementary Material

Supplementary Figures S1-S3

## Data Availability

All data are contained within the manuscript.

## Abbreviations

DTNB: 5,5′-dithiobis-(2-nitrobenzoic acid)
EPR: electron paramagnetic resonance
FMO: flavin monooxygenase
HEPES: 4-(2-hydroxyethyl)-1-piperazineethanesulfonic acid
HPLC: high-performance liquid chromatography
IPTG: isopropyl-β-D-thiogalactopyranoside
TMA: trimethylamine
TMAO: trimethylamine N-oxide
