# Supplementary material for: Two-component carnitine monooxygenase from Escherichia coli: functional characterization, inhibition and mutagenesis of the molecular interface
Source: Biosci Rep. 2022 Sep 21;42(9):BSR20221102. doi: 10.1042/BSR20221102 (PMC9508527; doi:10.1042/BSR20221102)
Supplement: Supplementary Figures S1-S3 [file BSR-2022-1102_supp.pdf]

Supplementary Information

Two-component carnitine monooxygenase from *Escherichia coli*: Functional characterization, Inhibition and mutagenesis of the molecular interface

Fabian Piskol[1], Kerstin Neubauer[1], Maurice Eggers[1], Lisa Margarete Bode[1], Jan Jasper[1], Alan Slusarenko[2], Edward Reijerse[3], Wolfgang Lubitz[3], Dieter Jahn[4] and Jürgen Moser[1]

[1]Institute of Microbiology, Technical University Braunschweig, Braunschweig, Germany
[2]Institut für Biologie III, RWTH Aachen University, Germany
[3]Max-Planck-Institute for Chemical Energy Conversion, Mülheim an der Ruhr, Germany
[4]Braunschweig Centre of Integrated Systems Biology, Braunschweig, Germany

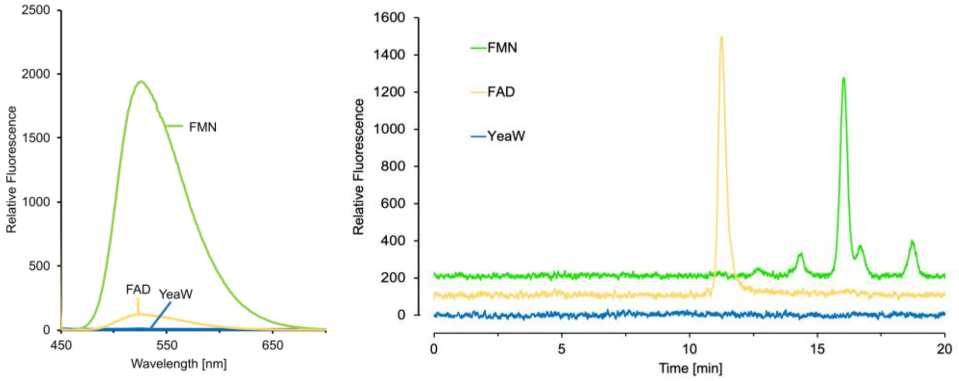

Figure S1. Fluorescence and HPLC analyses of YeaW. *left*, fluorescence measurement of a purified YeaW sample (370 nm excitation). Equimolar samples of an FMN or FAD standard were analyzed accordingly. *right*, a purified sample of YeaW (~50 µM) in a volume of 300 µl was incubated at 99 °C for 10 min. The resulting supernatant after centrifugation was chromatographed on a Reprosil 100 C18 column using excitation/emission wavelengths of 370/526 nm (*blue*). Authentic samples of FAD (*yellow*) or FMN (*green*) were analyzed accordingly.

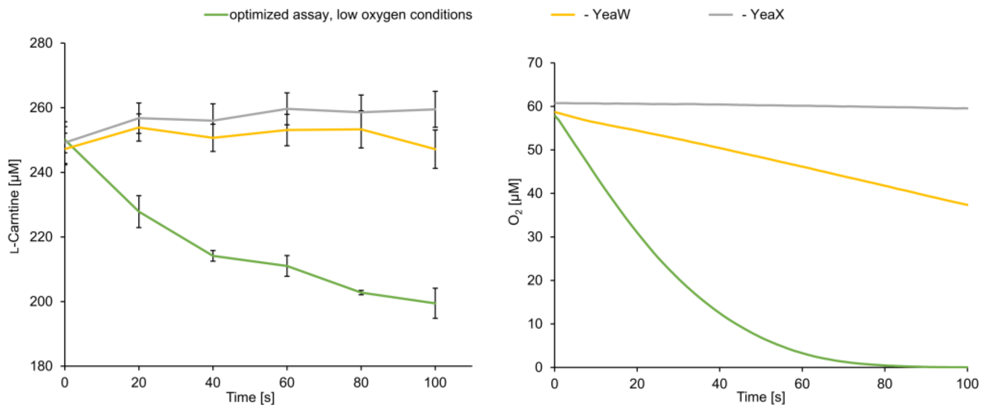

Figure S2. Carnitine monooxygenase activity under low oxygen conditions. The L-carnitine depletion assay and the O_2 depletion assay (*green traces*) were performed in an anaerobic chamber using N_2 saturated buffers. Mixtures of oxygen-free and air saturated assay components facilitated for the adjustment of a specific oxygen partial pressure of ~5%. A fiber-optic oxygen meter (FireSting, PyroScience) was used to validated the resulting oxygen values. Control reactions in the absence of YeaW (*yellow traces*) or YeaX (*grey traces*) were performed. *left*, L-carnitine depletion assay (prepared in triplicate, details under experimental procedures). *right*, continuous O_2 depletion assay. One representative graph from three measurements is shown.

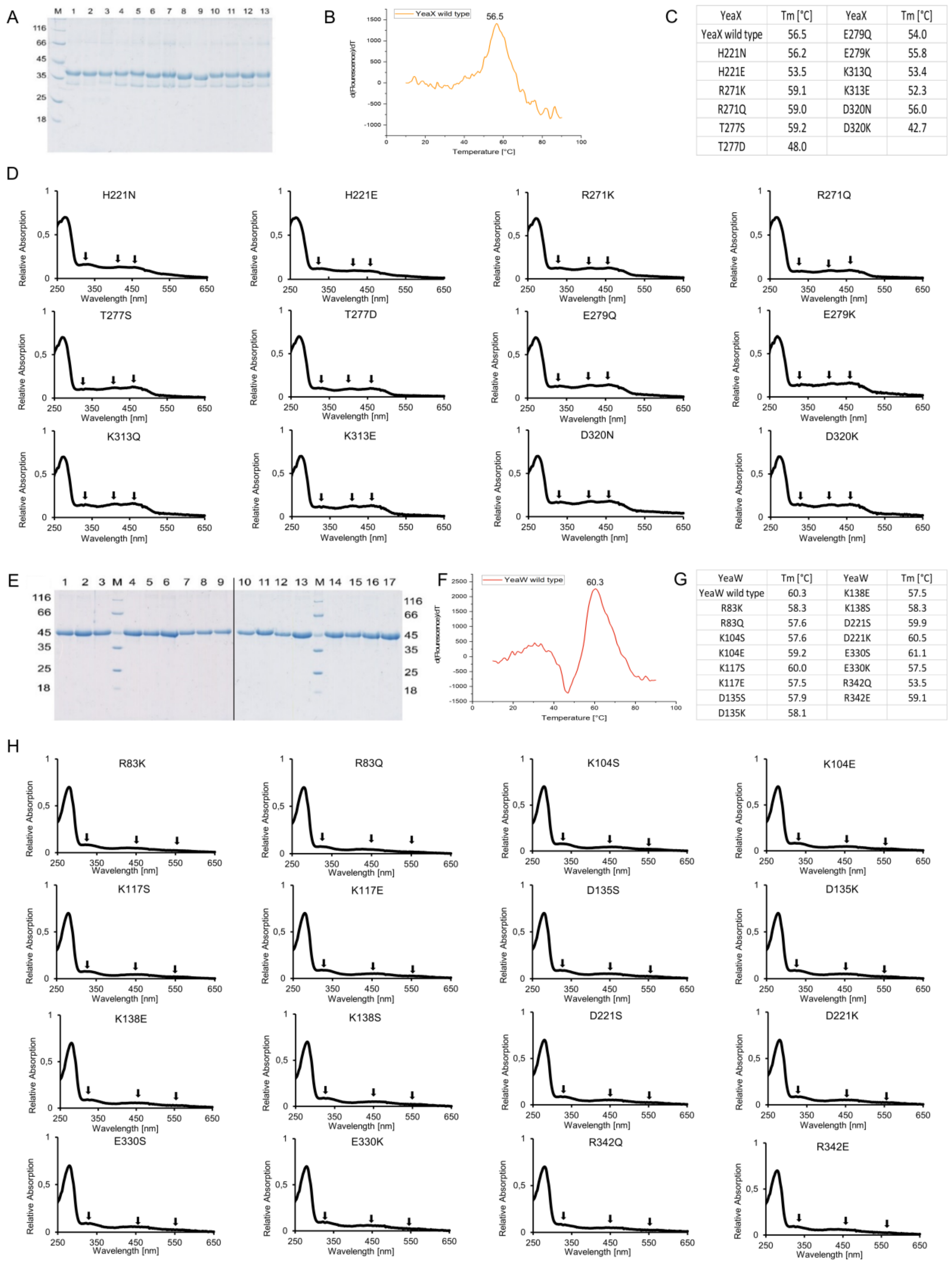

YeaX	Tm [°C]	YeaX	Tm [°C]
YeaX wild type	56.5	E279Q	54.0
H221N	56.2	E279K	55.8
H221E	53.5	K313Q	53.4
R271K	59.1	K313E	52.3
R271Q	59.0	D320N	56.0
T277S	59.2	D320K	42.7
T277D	48.0		

YeaW	Tm [°C]	YeaW	Tm [°C]
YeaW wild type	60.3	K138E	57.5
R83K	58.3	K138S	58.3
R83Q	57.6	D221S	59.9
K104S	57.6	D221K	60.5
K104E	59.2	E330S	61.1
K117S	60.0	E330K	57.5
K117E	57.5	R342Q	53.5
D135S	57.9	R342E	59.1
D135K	58.1		

Figure S3. Characterization of YeaX and YeaW proteins. A and **E**, SDS-PAGE analysis of purified YeaX mutants (*lanes 1-13*: wild type, H221N, H221E, R271K, R271Q, T277S, T277D, E279Q, E279K, K313Q, K313E, D320N and D320K) and of YeaW variants (*lanes 1-17*: wild type, R83K, R83Q, K104S, K104E, K117S, K117E, D135S, D135K, K138S, K138E, D221S, D221K, E330S, E330K, R342E and R342Q; lanes M: molecular mass marker, relative molecular masses (x 1,000) are indicated. **B** and **F**, thermal denaturation experiments (Thermofluor assay) for wild type YeaX and YeaW. **C** and **G**, melting temperature of variant proteins of YeaX and YeaW. **D** and **H**, Normalized UV-visible absorption spectra of purified YeaX and YeaW proteins.
